# Inflammatory Markers in Dysmenorrhea and Therapeutic Options

**DOI:** 10.3390/ijerph17041191

**Published:** 2020-02-13

**Authors:** Zofia Barcikowska, Elżbieta Rajkowska-Labon, Magdalena Emilia Grzybowska, Rita Hansdorfer-Korzon, Katarzyna Zorena

**Affiliations:** 1 Department of Immunobiology and Environment Microbiology, Medical University of Gdańsk, Dębinki 7, 80-211 Gdańsk, Poland; kzorena@gumed.edu.pl; 2 Department of Physical Therapy, Medical University of Gdańsk, Dębinki 7, 80-211 Gdańsk, Poland; erlabon@gumed.edu.pl (E.R.-L.); rita.hansdorfer-korzon@gumed.edu.pl (R.H.-K.); 3 Department of Gynecology, Gynecological Oncology and Gynecological Endocrinology, Medical University of Gdańsk, Smoluchowskiego 17, 80-214 Gdańsk, Poland

**Keywords:** women’s health, dysmenorrhea, inflammation markers, progesterone, treatment

## Abstract

Dysmenorrhea often significantly reduces the quality of women’s life and is still an important public health problem. Despite numerous studies, the pathomechanism of dysmenorrhea is not fully understood. Previous research indicates the complexity of biochemical reactions between the endocrine, vascular, and immune systems. Prostaglandins play a major role in the pathomechanism of dysmenorrhea. In contrast, cytokines and other proinflammatory factors in primary dysmenorrhea are less studied. In addition to the applied pharmacotherapy, more and more studies proving the effectiveness of non-pharmacological methods appear. Therefore, the present work contains a review of the latest research concerning factors involved in dysmenorrhea, as well as therapeutic options. In the literature search, authors used online databases, PubMed, and clinitrials.gov and browsed through individual gynecology, physiotherapy journals and books.

## 1. Introduction

Dysmenorrhea is the occurrence of severe lower abdominal pain in women during menstruation. The pain often has a cramping nature and may radiate to the thighs or lower spine. Lower abdominal pain may be accompanied by vomiting, headache, back pain, diarrhea, fatigue, etc. [1,2]. Dysmenorrhea is classified as primary and secondary. Primary dysmenorrhea is characterized as pain resulting from excessive, pathological uterine contraction, without palpable, in clinical examinations, lesions within the lesser pelvis [3]. Secondary dysmenorrhea is caused by acquired lesions in the smaller pelvis, which include endometriosis, chronic pelvic inflammation, uterine fibroids cervical stenosis, and anatomical and functional abnormalities of the reproductive organs [2,4]. Primary dysmenorrhea usually occurs only a year or two after the menarche. Menstrual pain begins a few hours before or at the time of the occurrence of the menstrual bleeding and lasts for 2–3 days. The pain is the strongest during the first 24–36 h of menstruation [1]. According to the WHO data, as many as 94% of young girls aged 10–20 and 8.8% of women aged 19–41 suffer from menstrual pains [5]. Dysmenorrhea often significantly reduces the quality of life and can even be a reason for absence from school or work [1]. The present review focuses on inflammatory markers in the menstrual cycle and therapeutic options for preventing menstrual pain.

### The Course of the Menstrual Cycle

The menstrual cycle is characterized by cyclic hormonal changes that are regulated by a complex feedback system on the hypothalamus–pituitary–gonadal axis [6]. The system involves orderly and sequential release from the pituitary of luteinizing hormone and follicle-stimulating hormone in response to gonadotropin-releasing hormone from the hypothalamus. This causes in the growth and maturation of follicles in the ovary, oocyte maturation, and estrogen and progesterone secretion [7]. Estrogen and progesterone are two main hormones secreted by the ovaries [6]. The menstrual cycle can be divided into several phases. The first half of the menstrual cycle consists of menstruation and the follicular phase during which an increase in estrogen concentration takes place. In the second half of the cycle, in the luteal phase, the peak of progesterone secretion occurs, and then, if fertilization did not happen, approximately 3 days before the onset of menstrual bleeding, the level of progesterone decreases [8]. In women, the menstrual cycle lasts 24–35 days, on average 28 days [7]. The onset of menstruation is associated with a decrease in the level of steroid hormones, progesterone and estradiol. When the egg cell is not fertilized, the corpus luteum responsible for the production of progesterone disappears and the level of the hormone decreases [9]. A lower level of progesterone causes a release of acid phosphatase and lytic enzymes present in lysosomes into the cytoplasm. These enzymes digest cells causing the release of prostaglandins. A decrease in progesterone also contributes to the inflammatory response that leads to exfoliation of the endometrium and menstrual bleeding [1]. The mechanisms that follow a decrease in progesterone concentration are complex reactions between the endocrine, vascular and immune systems [9].

## 2. Pathogenesis of Dysmenorrhea

Despite numerous studies, the pathomechanism of dysmenorrhea is not fully understood. Previous studies have shown that dysmenorrhea is a complex process that may depend on many factors [1,7,10]. It is known that the menstrual cycle is dependent on cyclic changes in ovarian hormone concentrations, and therefore also on cyclic changes in prostaglandin level and uterine contractile activity [11,12]. As early as in 1965, Pickles et al. noted that one of the factors contributing to dysmenorrhea may be an increase in prostaglandin concentration before menstruation [13]. These suggestions were confirmed in subsequent years by other authors who have demonstrated that prostaglandins are overproduced in dysmenorrhea [14]. This is also indicated by the symptoms that co-occur with dysmenorrhea during menstruation [3]. Prostaglandins cause narrowing of the blood vessels supplying the uterus, abnormal contractile activity of the uterus, which leads to ischemia, hypoxia of the uterus and increased sensitivity of the nerve endings [3,7,12]. In addition to hormonal changes that occur in the body, other factors, including diet, early age of the menarche, stress, length, and severity of menstrual periods, and the occurrence of premenstrual syndrome (PMS) may contribute to dysmenorrhea pathomechanism. The publications suggest the role of social, living, and psychological factors have been published [10,15,16,17]. Moreover, Finn proposed that menstruation could be regarded as an inflammatory event, because during menstruation the leukocytic invasion and subsequent production of inflammatory mediators is observed [18].

## 3. Uterus, Myometrium, Hormone Synthesis, and Inflammation

The mucous membrane of the uterus—the endometrium is the one of the most sensitive tissues to the hormones produced by the ovaries. It consists of two layers: a functional layer and a basal layer. The functional layer develops to allow implantation of the blastocysts, and then it undergoes exfoliation during menstruation. The basal layer is situated closest to the muscular layer of the uterus and its thickness is the same throughout the menstrual cycle. Its task is to rebuild the endometrium after exfoliation of the functional layer. The endometrium undergoes cyclic changes that can be divided into menstrual, proliferative, secretory phases, as well as a phase of preparations for embryo implantation and disintegrative one [19,20]. During the menstrual phase, when exfoliation of the endometrium occurs an essential role play metalloproteinases (MMPs). MMPs are enzymes produced by endometrial cells and leukocytes. The secretion of MMPs is probably inhibited by progesterone, which is why a decrease in progesterone causes an increase in MMP secretion [9]. Before menstruation, endometrial tissue acquires the characteristics of inflammation, it becomes red and edematous. Endometrial edema is the result of local increased production of chemokines including interleukin 8 (IL8), proinflammatory cytokines (IL1, IL6, TNFα) and leukocyte inflow [9]. The subsequent phase is the proliferative phase, which lasts until ovulation. During this time, an increase in estrogen concentration which induces endometrial proliferation occurs. After ovulation, both progesterone and estradiol affect the endometrium. In the secretory phase, progesterone level increases. Three days before the onset of monthly, progesterone and estradiol disappear, which initiates endometrial transformation: vasomotor reactions, apoptosis, tissue atrophy, and menstruation [21]. The myometrium is responsible for the expansion of the uterus during pregnancy and its contractility, which is especially important during delivery. The role of myometrium in the uterus of a non-pregnant woman consists in supporting the transport of spermatozoa to the Fallopian tube, preventing the penetration of microorganisms and removing infectious agents [22,23].

## 4. The Role of Progesterone in the Menstrual Cycle and the Development of Inflammation

**Figure 1:**
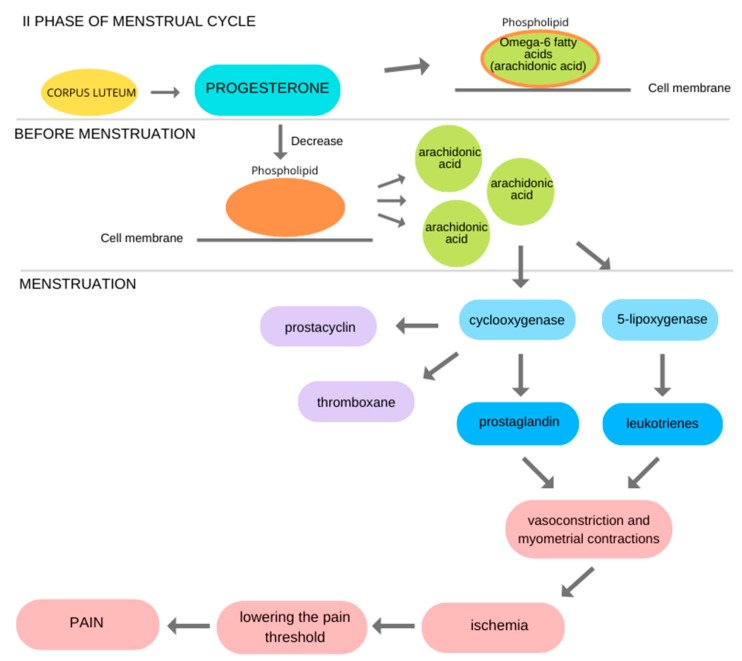
Possible mechanism of menstrual pain. Modified Figure 2 of [25].

Progesterone has anti-inflammatory effect, during the secretory phase it inhibits the release and activation of metalloproteinases. It also affects the regulation and synthesis of prostaglandins and leukocytes [9,14]. After ovulation, fatty acids accumulate in phospholipids in the cell membrane. Omega-6 fatty acid and arachidonic acid are released only when the level of progesterone begins to fall. The secretion of prostaglandins and leukotrienes, which causes uterine contractions, but also ailments such as vomiting, tympanites, nausea, and headache start. Arachidonic acid is metabolized by two pathways, cyclooxygenase and 5-lipoxygenase pathway. The first of them produces prostaglandins (PGF_2α_ and PGE_2_), prostacyclins and thromboxane. Leukotrienes are formed in the 5-lipoxygenase pathway. Arachidonic acid metabolites such as PGF_2α_ prostaglandin and cyclooxygenase cause vasoconstriction, uterine smooth muscle contraction leading to ischemia, lowering the pain threshold which results in pain [2]. Arachidonic acid through the action of cyclooxygenase COX-2 and lipoxygenase is a precursor in the production of prostaglandins, prostacyclins, thromboxane, and leukotrienes [24] (Figure 1).

## 5. The Role of Prostaglandins in Dysmenorrhea and the Development of Inflammation

It has been proven that prostaglandins are associated with inflammation and that they are produced during menstruation. The subject of research of scientists is also the impact of prostaglandins on the occurrence of pain during menstruation. Prostaglandin F_2α_ (PGF_2α)_ and Prostaglandin E_2_ (PGE_2_) have specific roles in the inflammatory process. PGF_2α_ mediates the constriction of arcuate vessels leading to local hypoxia of endometrial tissues. Another task of PGF_2α_ is to stimulate smooth muscle to contract, which in turn supports menstrual bleeding. The action of PGE_2_ depends on the type of receptors, but it can include the relaxation of endometrial blood vessels and may work to increase swelling and recruit leukotrienes [24]. In addition, prostaglandins may be involved in the formation of other chemokines and growth factors involved in the inflammatory response or in the repair process after menstruation. Prostaglandins may also increase the migration of neutrophils and leukocytes into the endometrium [24].

In a study by Lundstrom and Green in 1978, prostaglandin levels in women with menstrual pain versus healthy controls were compared [14]. They evaluated PGF_2α_ and its metabolites in plasma and endometrium. Plasma samples were taken on the first day of menstruation, while endometrial biopsy was performed before menstruation and on the first day of menstruation. They obtained a significant difference in the plasma concentration of the metabolite of PGF_2α_ prostaglandin between the group of women without painful menstruation and the pain group. Additionally, a significantly higher concentration of prostaglandins was demonstrated in the endometrium in women suffering from pain. However, before menstruation a similar difference was not obtained when assessing the concentration of prostaglandins in the endometrium. The authors also described correlations of uterine contractions with prostaglandins level. Women with a high level of prostaglandins in both endometrium and plasma were observed, they experienced severe uterine contractions [14]. Similar results were obtained by Stromberg et al., who studied the level of vasopressin and the metabolites of prostaglandins in women who had premenstrual pain or dysmenorrhea compared to group of women without any pain [26]. The study included 20 women, who were divided into four groups. The first group consisted of women who felt lower abdominal pain 1–3 days before menstruation. Moreover, these women often experienced swelling, changes of mood and pain during menstruation. The second group consisted of women who did not experience any symptoms of premenstrual syndrome. The third group were women whose main problem was the occurrence of dysmenorrhea with accompanying symptoms such as acute pain, vomiting, and nausea. The last group was constituted by women without dysmenorrhea. The authors demonstrated that women with premenstrual syndrome symptoms have higher vasopressin levels. Similarly, they found no significant difference in the level of prostaglandin metabolites. In the group with dysmenorrhea, they showed that both the concentration of vasopressin and prostaglandin metabolites is statistically significantly higher in women with dysmenorrhea than in women without pain [26]. The level of PGF_2α_ prostaglandin metabolites have also been studied by Liedman et al. however, they did not demonstrate a significant difference between women who suffered from dysmenorrhea and women who menstruated painlessly [27]. PGF_2α_ and PGE_2_, produced by myometrium, also participate in the synthesis of many other proteins, which include cytokines [22].

## 6. Vasopressin

Vasopressin is a hormone secreted by the pituitary gland, its secretion is stimulated by cyclic changes in estradiol concentration. The concentration of vasopressin is lower in the follicular phase, then it increases during ovulation. Vasopressin may contribute to an increase in uterine contractile activity and reduce blood flow through the uterus, which in turn may lead to the ischemia and occurrence of dysmenorrhea [26,28,29]. The role of vasopressin in dysmenorrhea pathomechanism is emphasized by the several authors [26,27,29]. In the study, it has been demonstrated that during ovulation vasopressin levels were lower in women with dysmenorrhea than in healthy women, while during menstruation they did not observe significant changes [27]. In the examination of women who suffer from premenstrual syndrome or dysmenorrhea, it was found that the concentration of vasopressin is higher compared to women without similar symptoms [26]. However, in other studies that do not confirm the role of vasopressin in dysmenorrhea the authors compared the concentration of vasopressin in women suffering from dysmenorrhea and healthy women. The results did not show a significant difference in concentration between the two groups [30].

## 7. Cytokines and Other Proinflammatory Factors as Inflammatory Mediators in Primary Dysmenorrhea

Cytokines are mediators of the body’s immune response and are involved in the regulation of reproductive processes, the menstrual cycle and pregnancy [22,23,31]. The role of cytokines was emphasized in the embryo implantation process, which determines the maintenance and normal course of pregnancy. In the absence of pregnancy, cytokines participate in cyclic transformations of uterine tissues [32]. The changes in the concentration of inflammatory factors during the menstrual cycle are shown in Table 1.

### 7.1. Tumour Necrosis Factors Alpha (TNFα)

Activated macrophages produce proinflammatory cytokines TNFα, IL1, IL6, etc. responsible for upregulating inflammatory responses [33,34]. It has also been reported that these mediators stimulate the synthesis or release of prostaglandins [23,31,34], causing excessive contraction of the uterine muscle, which leads to ischemic pain of primary dysmenorrhea. Plasma IL6 and TNFα levels were found to be higher in women with dysmenorrhea compared to women without menstrual disorders [33]. Furthermore, TNFα is a cytokine that is responsible for inhibiting endometrial proliferation and induces apoptosis. Previous studies have shown that endometrial cells produce increased concentration of TNFα during menstruation [35]. Moreover, Dogru et al. showed that the TNF -308G > A gene polymorphism is strongly associated with susceptibility to dysmenorrhea in the Turkish female population [36]. The authors demonstrated that the presence of the -308A TNFα allele can protect against dysmenorrhea and suggested that the TNFα-308, GG genotype may be a useful tool for predicting susceptibility to dysmenorrhea [36]. Recent studies have shown that intensive aerobic exercises not only cause a reduction in the levels of the metabolite of prostaglandins (13,14-dihydro-15-ketoprostaglandin F2 alpha (KDPGF_2α_), but also reduce the level of TNFα, as well as reduce the intensity of pain associated with dysmenorrhea [34].

### 7.2. Interleukin 6 (IL6) in the Menstrual Cycle

IL6 is a pleiotropic cytokine with multi-directional effects on the cells of the innate and acquired immunity system. The main role of IL6 is to initiate and regulate the acute inflammatory response and to facilitate the development and targeting of the acquired response. IL6 exhibits pro- and anti-inflammatory properties and is now considered an important target for clinical intervention [37]. In a study of women with a normal menstrual cycle, significant variability in plasma cytokine levels, including IL1β, IL6, IL8, and IL10 were observed [38]. Levels of several factors increased during ovulation and then achieved their peak during menstruation, which is considered by some scientists to be a proinflammatory event [21]. Levels of IL1β, IL6, and IL8 were inversely correlated with estradiol and progesterone levels (*P* < 0.01), further supporting immune involvement in the menstrual cycle [39]. However, Angstwurm et al. demonstrated an increase in IL6 concentration in the follicular phase when the level of estradiol increased. After ovulation, in the luteal phase, when there was a 10-fold increase in progesterone, the level of IL6 in plasma decreased 1.5–4.4 times. In the subsequent cycle, IL6 level increased again [40]. Increased IL6 concentration in women with dysmenorrhea has also been shown in other studies [41,42]. The authors demonstrated that the level of IL6 was statistically significantly higher during the luteal phase compared to the follicular phase [42].

### 7.3. Vascular Endothelial Growth Factor (VEGF)

Few studies indicate the involvement of VEGF in the process of dysmenorrhea among women with endometriosis [43,44]. It is known that VEGF is the strongest factor involved in the embryonic development, menstrual cycle, and in ovarian endometriomas [45]. Although VEGF is produced by cells and tissues of the reproductive tract, such as the endometrium, ovary, and placenta, VEGF receptors are found only in the endothelial cells. VEGF has been shown to stimulate endothelial cell proliferation, migration and increase vascular permeability [46]. Recent reports have shown a relationship between the production of VEGF, macrophage migration inhibitory factor (MMIF), hypoxia-inducible factor-1 a (HIF-1α) and the stage of endometriosis, as well as the severity of dysmenorrhea [43]. The expression of all three proteins in endometrial tissues and in serum increased significantly with the severity of pain (*P* < 0.05). Thus, the authors conclude that MMIF, HIF-1α and VEGF expression in serum can be used to assess the stage of endometriosis, as well as the severity of dysmenorrhea [43].

### 7.4. C-Reactive Protein (CRP) in the Menstrual Cycle

CRP is a clinically recognized acute phase protein. It is assumed that normal CRP concentration in healthy people should not exceed 3 mg/L. During the acute phase reaction, which is a defense response to inflammation, infection or injury, the concentration of serum CRP can increase up to 1000-fold, reaching its maximum concentration after 24–48 h [47,48]. CRP is an important marker of the ongoing inflammatory response, with a relatively short half-life (~48 h), its concentration returns to its baseline in 7 to 12 days [49]. CRP also supports the process of phagocytosis, affecting monocytes, macrophages, and neutrophils, as well as acting in a chemotactic and opsonizing way [50]. In addition, it induces monocytes/macrophages to synthesize pro-inflammatory cytokines and inhibits the synthesis of anti-inflammatory cytokines. Studies conducted in adult women have shown that increased levels of CRP varied significantly across the menstrual cycle. CRP was highest during menses, decreased during the follicular phase, was lowest on the expected day of ovulation, and increased in the luteal phase [51]. Another study, showed that a ten-fold increase in progesterone was associated with a 23% increase in CRP (*P* = 0.01), a ten-fold increase in estrogen was associated with a 29% decrease in CRP (*P* = 0.05) [52]. In a study, with the participation of healthy women, CRP levels were positively correlated with the severity of menstrual symptoms, the strongest being mood and pain symptoms [53].

## 8. Pharmacological Treatment Options for Menstrual Pain

Non-steroid anti-inflammatory drugs (NSAIDs) are a first-line treatment in dysmenorrhea. The development of NSAIDs, in 1969, started a new era of pain management, whilst making anti-inflammatory drugs available over the counter provided women with the prospect of reducing menstrual pains [54]. NSAIDs act by inhibiting cyclo-oxygenase (COX), which is the enzyme responsible for the synthesis of prostaglandins [25,54,55,56]. It has been demonstrated that a reduced production of prostaglandins decreases the strength of uterine contractions, thus relieving women’s discomfort [25]. Two forms of COX, COX-1, and COX-2 have been identified to date [55]. Suppressing both COX-1 and COX-2 activity, traditional NSAIDs such as ibuprofen, naproxen, diclofenac potassium, and meclofenamate are non-selective inhibitors [7,55]. A decrease in the production of prostaglandins was revealed in women with dysmenorrhea who had been administered with naproxen sodium. Consequently, uterine contractions and pain were reduced [14]. Studies demonstrated that NSAID treatment is the most effective if it starts 1–2 days before the beginning of the menstruation [7,25]. It has also been revealed that NSAIDs are more effective than placebo in women with dysmenorrhea, and that they were significantly more likely to cause adverse reactions [1,55]. The failure rate of NSAIDs treatment is estimated to be 20%–25%, whereas some drugs may be contraindicated and may not be tolerated by women [54]. NSAIDs are weak acids and as such can induce damage to the stomach mucosa, cause gastric erosion, ulcers, as well as gastrointestinal bleeding. Studies to date have revealed that while taking NSAIDs, female patients, just from the beginning, can experience nausea, dyspepsia, headaches, dizziness, drowsiness, or dry mouth [55,56]. Moreover, NSAIDs can have a negative effect on the kidneys, liver and the circulatory system, increasing the risk of thromboembolic complications [54,56]. Authors, however, point to the fact that dysmenorrhea lasts only 2–3 days, which reduces the risk of adverse effects caused by NSAIDs [25].

Importantly, hormone therapy for at least three menstrual cycles could be administered to women whose dysmenorrhea is not reducible by NSAIDs [25]. Therefore, the second possibility to treat menstrual pain lies in the use of hormonal contraceptives, and combined oral contraceptives in particular. The aforementioned therapy combines estrogens and progestogens [57]. Combined hormonal contraceptives, including oral contraceptives, contraceptive rings, and patches act by limiting the growth of endometrium. Endometrium produces prostaglandins and leukotrienes that contribute to the development of dysmenorrhea. The role of hormonal contraceptives also consists in inhibiting ovulation and, consequently, progesterone production, which also reduces the synthesis of prostaglandins and leukotrienes [7,25]. Lower doses of hormonal contraceptives are being currently used, which reduces the risk of adverse reactions. However, such doses may still predispose to breast cancer or venous thrombosis [58,59]. It has also been proved that long-acting reversible contraceptives are an effective method for treating dysmenorrhea. Levonogestrel intrauterine system (LNG-IUS), a subcutaneous implant containing etonogestrel as well as depot medroxyprogesterone acetate (DMPA) are such contraceptives [7]. The findings of the study by Suhonen et al. [60] demonstrated greater efficacy of LNG-IUS in comparison with combined oral contraceptives. It was also indicated that a subcutaneous implant containing etonogestrel was of similar efficacy to LNG-IUS, accompanied by a reported 81% improvement in dysmenorrhea [61]. The researchers identified endometrial atrophy caused by LNG-IUS, inhibition of the ovulation caused by depot medroxyprogesterone acetate as well as the etonogestrel implant as mechanisms responsible for the beneficial effect on dysmenorrhea [7,60,61]. An analgesic effect of long-acting reversible contraceptives in women with dysmenorrhea can be explained by endometrial atrophy due to LNG-IUS and inhibition of ovulation caused by depot medroxyprogesterone acetate and an etonogestrel implant [7]. Hormonal therapy may be a first-line treatment in sexually active dysmenorrhea patients [25]. 

Calcium channel blockers are another group of drugs investigated in relation to dysmenorrhea treatment [54,62,63,64,65]. In general, calcium channel blockers are primarily indicated for the treatment of hypertension [62,63]. However, by reducing contractility of the vascular smooth muscles they also inhibit uterine contractions [63]. The studies to date have demonstrated that administering 20–40 mg of nifedipine, of blocking calcium channels properties to women provided them with a relief from menstrual pain, but was also associated with such adverse effects as tachycardia, hot flushes and headaches [64,65].

Antagonists for vasopressin and oxytocin receptors are the next group of drugs investigated in the treatment of menstrual pain. Vasopressin and oxytocin, hormones stimulating myometrial contractions, are also related to primary dysmenorrhea [27,66,67]. It has been indicated that vasopressin-evoked contractions in women with dysmenorrhea may be reduced by 1-deamino-2-d-Tyr(OEt)-4-Thr-8-Orn-oxytocin (Atosiban) [68] as well as by orally administered SR49059 [69]. Conflicting findings were presented by Valentin et al. [30] who did not demonstrate an increased level of vasopressin in women with dysmenorrhea in comparison to healthy women. Intravenous administration of atosiban to women with dysmenorrhea did not reduce menstrual pain nor uterine contractility in comparison to the healthy control group [30]. Naloxone hydrochloride also has a significant role in the regulation of vasopressin and oxytocin release from the posterior lobe of pituitary gland [67]. Naloxone hydrochloride is the best known antagonist for opioid receptors able to specifically block vasopressin and oxytocin. Ten women diagnosed with dysmenorrhea took part in one of the studies [67]. Spontaneous contractile activity of the uterus was recorded for 30 min. on the first day of menstruation. Subsequently, naloxone was administered intravenously and intrauterine pressure was recorded on for 2 h. The records of intrauterine pressure at dysmenorrhea, demonstrated enhanced spontaneous contractile activity, characterized by high frequency and contraction amplitude. Intravenous administration of naloxone had a positive effect on limiting contractile activity of the uterus. These changes correlated with a decrease of menstrual pain experienced by female patients with primary dysmenorrhea [67]. 

## 9. Physiotherapy for Dysmenorrhea

Other methods used to reduce the discomfort of dysmenorrhea are acupuncture, yoga, massages, as well as physiotherapy. However, their effectiveness has not been consistently verified in large randomized controlled trials [70,71,72,73]. The method whose efficacy in relieving dysmenorrhea was confirmed in several randomized trials is transcutaneous electrical nerve stimulation (TENS) [74]. The issue of physiotherapy, and the possibility of effective use of manual techniques in painful menstruation was examined by researchers Barcikowska et al. [75]. Through the use of post-isometric relaxation and trigger point therapy of selected muscles that attach to the pelvis it has been shown that the menstrual pain was reduced in young women with dysmenorrhea [75]. After manual therapy, in each woman the decrease of progesterone level was observed [75]. Other authors also investigated the effectiveness of connective tissue manipulation [76]. The patients were divided into the experimental group and the control group. Each of these groups had healthy habits and stretching exercises recommended. In addition, connective tissue manipulations were performed in the experimental group. The researchers achieved a statistically significant improvement in the experimental group, while in the control group a deterioration was obtained. In another study that also demonstrated the effectiveness of manual techniques in patients with dysmenorrhea, authors compared the effectiveness of neuromuscular techniques to pharmacotherapy [77]. According to a study conducted by Molins-Cubero et al. the pelvic manipulation is effective in case of lower back pain in women with painful menstruations [77]. The study included 40 women, who were dichotomized into, the control and the study groups. In the study, they used the visual scale of pain sensation and tests with dynamometer of the lowest pressure value that causes discomfort and pain. In addition, they checked whether the therapy could affect the secretion of catecholamines (noradrenaline, adrenaline, dopamine) and serotonin. After the therapy, a statistically significant difference between the control group and the study group, was obtained only for serotonin concentration and for the level of pain sensation. The authors did not observe statistically significant changes in adrenaline and noradrenaline concentration [78]. Another study that assessed the effectiveness of manual techniques, namely spine manipulation, is research conducted by Kokjohn et al. as early as in 1992 [79]. They examined the level of pain experienced by patients and the level of the prostaglandin metabolite. The significant relief of pain in the experimental group was observed, however a decrease in the concentration of the prostaglandin metabolite was reported in both the experimental group and the control group [79]. There are more and more studies demonstrating the possibility of using physiotherapy in case of dysmenorrhea [80,81,82]. Well known and a widely investigated form of therapy is the use of TENS [80]. Simple devices with programs that allow patients for independent TENS current therapy are available [83]. Recent reports also inform about the possibility of using treatments with the use of high-energy laser radiation and pulsed magnetic field. The authors compared the effect of both methods on reducing pain and assessed the impact of the studied physical stimuli on the concentration of PGF_2α_ prostaglandins. In both groups, they obtained a statistically significant reduction of pain and PGF_2α_, while in a comparison of the effectiveness of therapy between groups, the treatment with high-energy laser radiation was more effective [81]. Researchers also attempt to determine the effect of various forms of exercise on reducing dysmenorrhea symptoms. The effectiveness of a special developed comprehensive program consisting of stretching exercises, pelvic floor muscle exercises, jogging, and short relaxation was proved [73]. In turn, others, demonstrated that isometric exercises of pelvic floor muscles, abdominal muscles, hip abductor muscles may reduce dysmenorrhea symptoms [72]. The authors Motahari-Tabari et al. studied the use of abdominal and pelvic muscle stretching compared to the use of mefenamic acid, a medication from the NSAID group. It is interesting that they found no statistically significant differences between the applied therapies [84]. Yoga can also be used to relieve pain. There are studies reporting the effective use of selected yoga positions to reduce discomfort [71,85,86]. In addition, Chien et al. have proven that yoga can lower homocysteine level, which can probably be involved in dysmenorrhea pathogenesis [87]. The recent study investigated the effect of intensive exercise on dysmenorrhea alleviation as well as TNFα levels, prostaglandin metabolites, progesterone and selected interleukins [34]. Researchers have detected an increase in progesterone, and a decrease in prostaglandin metabolites and TNFα. The results they received may suggest that increasing the level of progesterone may lead to a decrease in prostaglandin production, which in turn will reduce pain [34]. Another, often described, effective method in alleviating dysmenorrhea is acupuncture. The effects of acupuncture with hormonal OC were compared and eventually very similar effects in both therapies were obtained [88]. Different methods that have been tested so far include selected techniques from the field of osteopathy, the application of kinesiotaping, aromatherapy and heat [89,90,91,92].

## 10. Conclusions

In the above review, selected inflammatory factors that are involved in dysmenorrhea, have been discussed. The methods, which may serve to alleviate menstrual pain have also been presented. There are numerous methods to reduce dysmenorrhea, but many of them require confirmation in more research. It is also justified to study the mechanisms of the diverse therapies such as physical activity, manual therapy, or physiotherapy, and their impact on the inflammatory factors in the pathogenesis of dysmenorrhea. A better understanding of the causes of dysmenorrhea may result in effective therapy and thus increase the comfort of living for thousands of women around the world.

## Figures and Tables

**Table 1:** Changes in the concentration of inflammatory factors during menstrual cycle.

Factors	Authors	Changes in the Concentration of Inflammatory Factors
**PGF_2α_**	Lundstrom and Green [14]	higher concentration during menstruation among women with dysmenorrhea
	Stromberg et al. [26]	higher in women with dysmenorrhea than in women without pain during menstruation
	Liedman et al. [27]	no difference between dysmenorrheic women and women without pain
**Vasopressin**	Stromberg et al. [26]	higher in women with dysmenorrhea than in women without pain during menstruation
	Liedman et al. [29]	lower during ovulation in women with dysmenorrhea than in healthy women, during menstruation without changes
	Valentin et al. [30]	absence of difference in concentration between group with dysmenorrhea and healthy women
**TNFα**	Ma et. al. [33]	increase in genes encoding pro-inflammatory cytokines TNFα
**IL6**	Angstwurm et al. [40]	increase in the follicular phase, decrease in the luteal phase
	Yeh et. al. [41]	higher in the dysmenorrhea women than in the healthy women
**VEGF**	Zhang F et al. [43]	the expression of VEGF in the serum and endometrial tissues may be used to assess the stage of endometriosis and the severity of dysmenorrhea.
	Xu H et al. [44]	supplementation and over-expression of VEGF-C significantly reversed the inhibitory effects on the endothelial functions, vascular permeability and endometriotic growth
**CRP**	Gaskins et. al. [51]	highest during menses, decrease during the follicular phase, lowest on the expected day of ovulation, and increase in the luteal phase

**Abbreviations:** PGF_2α_—prostaglandin F_2α_; TNFα—Tumor Necrosis Factorα; IL6—Interleukin 6; VEGF—Vascular Endothelial Growth Factor; CRP—C-Reactive Protein.
